# The undifferentiated carcinoma that became a melanoma: Re-biopsy of a cancer of an unknown primary site: a case report

**DOI:** 10.1186/s13256-017-1238-y

**Published:** 2017-03-27

**Authors:** Oluf Dimitri Røe, Sissel Gyrid Freim Wahl

**Affiliations:** 1 Cancer Clinic, Department of Surgery, Levanger Hospital, Nord-Trøndelag Hospital Trust, Kirkegata 2, N-7600 Levanger, Norway; 2 Department of Cancer Research and Molecular Medicine, Faculty of Medicine, Norwegian University of Science and Technology (NTNU), Prinsesse Kristinsgt. 1, N-7491 Trondheim, Norway; 3 Clinical Cancer Research Center, Department of Clinical Medicine, Aalborg University Hospital, Hobrovej 18-22, DK-9100 Aalborg, Denmark; 4 Department of Pathology and Medical Genetics, St. Olavs Hospital, Erling Skjalgssons gt. 1, N-7491 Trondheim, Norway

**Keywords:** Case report, Cancer of unknown primary, BRAF mutation, Melanoma, Immunotherapy

## Abstract

**Background:**

Cancer of unknown primary site is still a demanding condition as it is per definition metastatic, with heterogeneous biological behavior, and it is often resistant to therapy. Cancer of unknown primary site accounts for approximately 1 to 5 % of all cancers, but is currently among the top six causes of cancer deaths in Western countries. To correctly identify the biological origin of the tumor, a large spectrum of differential diagnoses must be considered and scrutinized. At progression, re-biopsy might be necessary to reveal the true origin of the tumor or actionable targets.

**Case presentation:**

A 62-year-old Norwegian woman, with a fast growing lump in her left groin, was primarily diagnosed as having undifferentiated carcinoma that was BRAF V600 positive. There was complete response with paclitaxel-carboplatin and she was recurrence-free for 18 months. She had recurrence in both lungs and subcutaneously in her left groin and thigh; a re-biopsy revealed transformation to a malignant melanoma. She was resistant to BRAF inhibitors, then treated with ipilimumab and is currently a long-term survivor of 4 years and 4 months since the first diagnosis, with no clinical or radiological evidence of recurrence.

**Conclusions:**

A biopsy from patients with metastasis of unknown primary should be analyzed thoroughly to identify organ of origin, molecular make-up, and possible molecular targets. Re-biopsy of cancer of unknown primary site at progression can reveal the true cellular origin of the tumor as well as provide novel therapeutic opportunities, including immunotherapy.

## Background

Cancer of unknown primary site (CUP) accounts for approximately 1 to 5 % of all cancers but has a dismal prognosis of approximately 10 to 20 % 1-year survival and in Australia is the sixth cause of cancer death [1–4]. Depending on the patient series, up to 40 % of patients with CUP are diagnosed as having metastasis in their lymph nodes, while the remaining patients present with metastasis in their internal organs [5, 6]. In a large series of CUP in various anatomical regions, patients with an affected lymph node showed a wide survival span; patients presenting with affected inguinal lymph nodes had a median survival of 18 months and patients affected intra-abdominally had a median survival of only 4 months [7]. Thus CUP is a very heterogenous entity, where histopathological examination, immunohistochemical profiling, and molecular profiling of the tumor are necessary to improve treatment and survival of patients with these tumors.

The complexity and unusual features of the history of this 62-year-old woman with a fast-growing mass in her left inguinal area, has taught us a lesson that may be important to share with oncologists treating this patient population.

## Case presentation

**Fig. 1:**
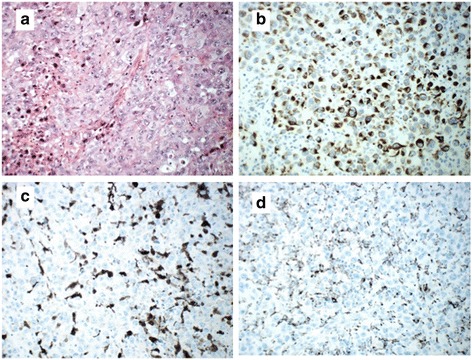
Tumor resected in 2012. The resection revealed malignant epithelioid cells with pale eosinophilic cytoplasm and pleomorphic nuclei with vesicular chromatin; hematoxylin, eosin and saffron staining (**a**). The resection revealed a positive reaction for cytokeratin AE1/AE3 (**b**). Scattered cells stained positive for S100 (**c**) and CD68 (**d**), representing tumor-associated macrophages, but tumor cells were S100 negative

A 62-year-old woman of Norwegian ethnicity, previously healthy, presented in September 2012 with a large fast-growing nodal mass in her left groin; a computed tomography (CT) scan showed a 62 mm tumor infiltrating the subcutis. A clinical examination did not reveal any other pathological glands or any skin lesions. An magnetic resonance imaging (MRI) of her pelvis showed a solitary tumor, and subsequent CT of her thorax and abdomen, mammography, and endoscopy could not reveal or indicate the primary location of the tumor; in addition, the tumor markers we use in our screening were all within normal range: carcinoembryonic antigen (CEA), CA125, CA15-3, CA19-9, neuron-specific enolase (NSE), and chromogranin A (CgA). In November 2012 the tumor was still regarded as operable and surgery was performed. On macroscopic examination, the tumor was a 57 mm solid tumor. Histopathological examination revealed a tumor composed of malignant epithelioid cells, which on immunohistochemical examination stained positive for cytokeratin (CK) AE1/AE3 (diffuse), CK7 (focally), CD10, vimentin, and epithelial membrane antigen (EMA; scattered tumor cells). S100 was positive in scattered cells with dendritic features and possibly in a few scattered tumor cells. Nearly 100 % of the tumor cells were positive for Ki67/MIB1. The cells were negative for BerEP4, CK20, CK5/6, p63, thyroid transcription factor 1 (TTF1), synaptophysin, chromogranin, estrogen and progesterone receptors, human melanoma black 45 (HMB45), melan A, leukocyte common antigen (LCA), desmin, myogenin, smooth muscle actin (SMA), and CD30 (Fig. 1). We screened for actionable targets and molecular genetic analysis was negative for *KRAS* and synovial sarcoma marker, translocation t(X;18), but revealed BRAF V600E mutation. In the final pathology report, the tumor was classified as a metastasis from an undifferentiated carcinoma.

**Fig. 2:**
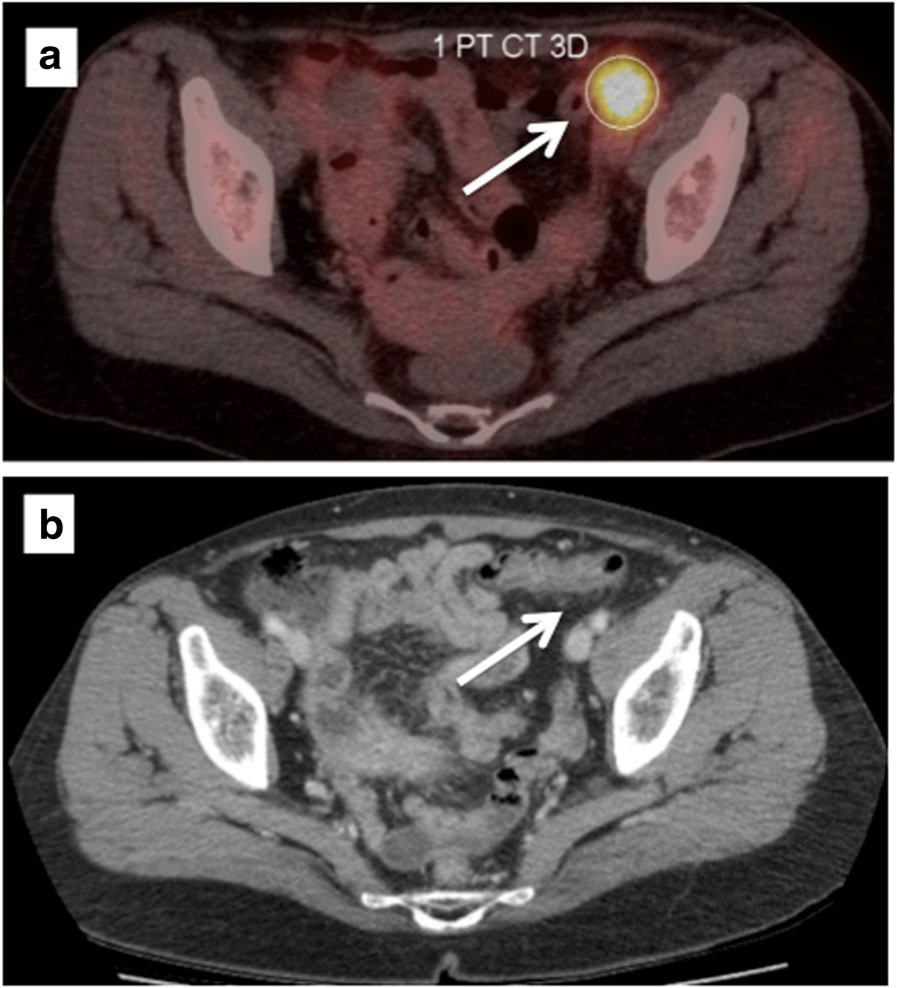
The patient had postoperative complications with infection and lymphatic leakage. Subsequent positron emission tomography-computed tomography (**a**) showed masses to be growing deeper in the pelvis, which could not be removed surgically (*white arrow*). Computed tomography scanning (**b**) showed complete remission after four courses of paclitaxel and carboplatin (*white arrow*)

Our patient had postoperative complications with infection and lymphatic leakage, subsequent CT scanning and positron emission tomography (PET)-CT showed masses to be growing deeper in her pelvis, which could not be removed (Fig. 2).

**Fig. 3:**
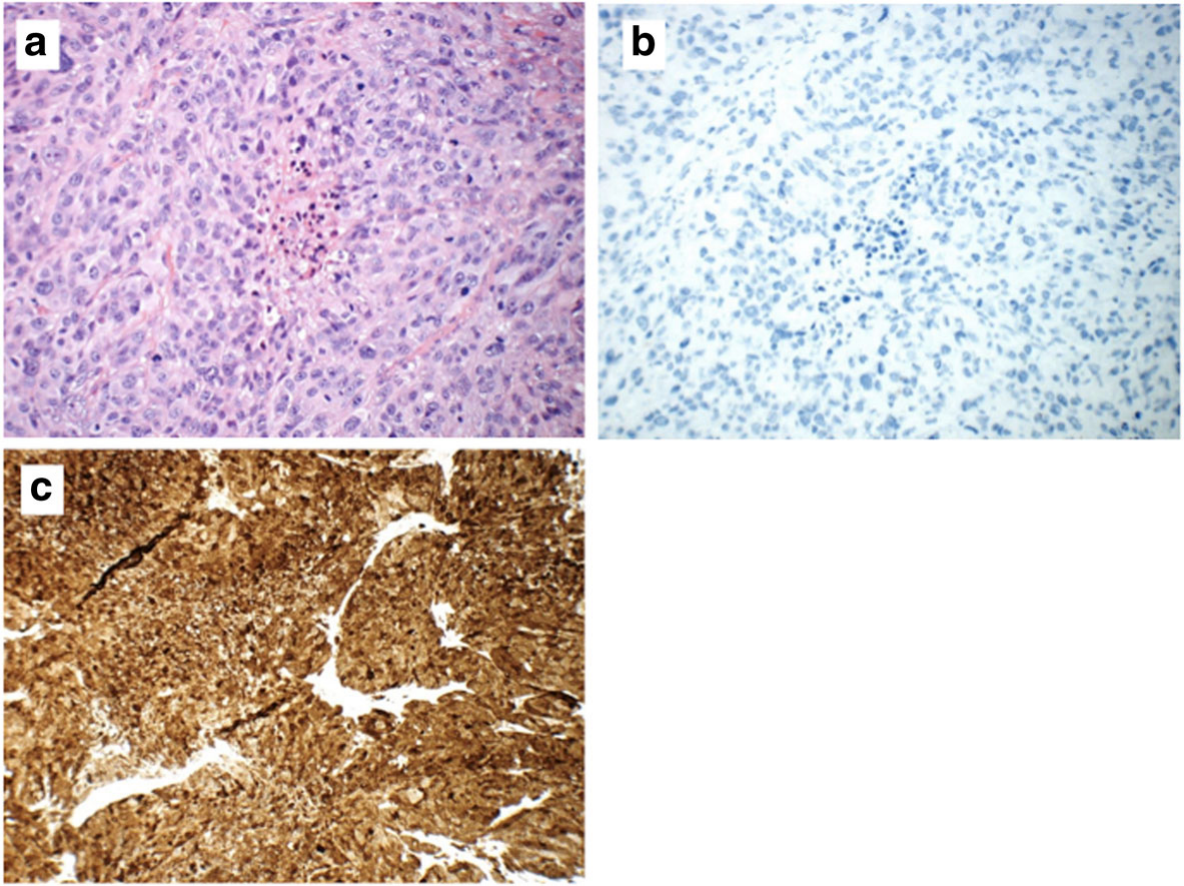
Several subcutaneous recurrent tumors developed after 18 months. A biopsy showed cells with a growth pattern, cellular features, and nuclear features similar to the tumor resected in 2012 seen by hematoxylin, eosin and saffron staining (**a**), but a change to negative staining for cytokeratin AE1/AE3 (**b**) and now positive staining for S100 (**c**)

We decided to give paclitaxel with carboplatin (AUC5) every 3 weeks, which is a standard treatment for CUP and after four courses the masses went into complete remission. Consolidation radiotherapy was performed April to May 2013, 2×(25 to 50) Gy. Our patient was in very good general condition during all the treatment period: World Health Organization (WHO) performance status (PS) grade 0. The remission lasted for 18 months to July 2014, when multiple, fast-growing subcutaneous nodules evolved within and near the surgical wound and radiation field and distally on her left thigh. Moreover, multiple small metastases were seen in both her lungs. One subcutaneous nodule was extirpated for histopathological analysis, which revealed a tumor with a cellular morphology and cellular growth pattern similar to the tumor resected 18 months earlier. The tumor cells were, as previously, negative for BerEP4, CK20, CK5/6, P63, HMB45 and melan A, but surprisingly CK AE1/AE3 was now negative and S100 strongly positive in all tumor cells (Fig. 3). The tumor cells were closely studied, both initially when the first report was made and on reevaluation, and conspicuous pigment was not detected.

The BRAF V600E mutation persisted, strongly indicating that the tumor was of the same origin but probably another clone, or a transformation from the primary tumor after chemoradiation with an unusual or aberrant expression profile for a melanoma.

**Fig. 4:**
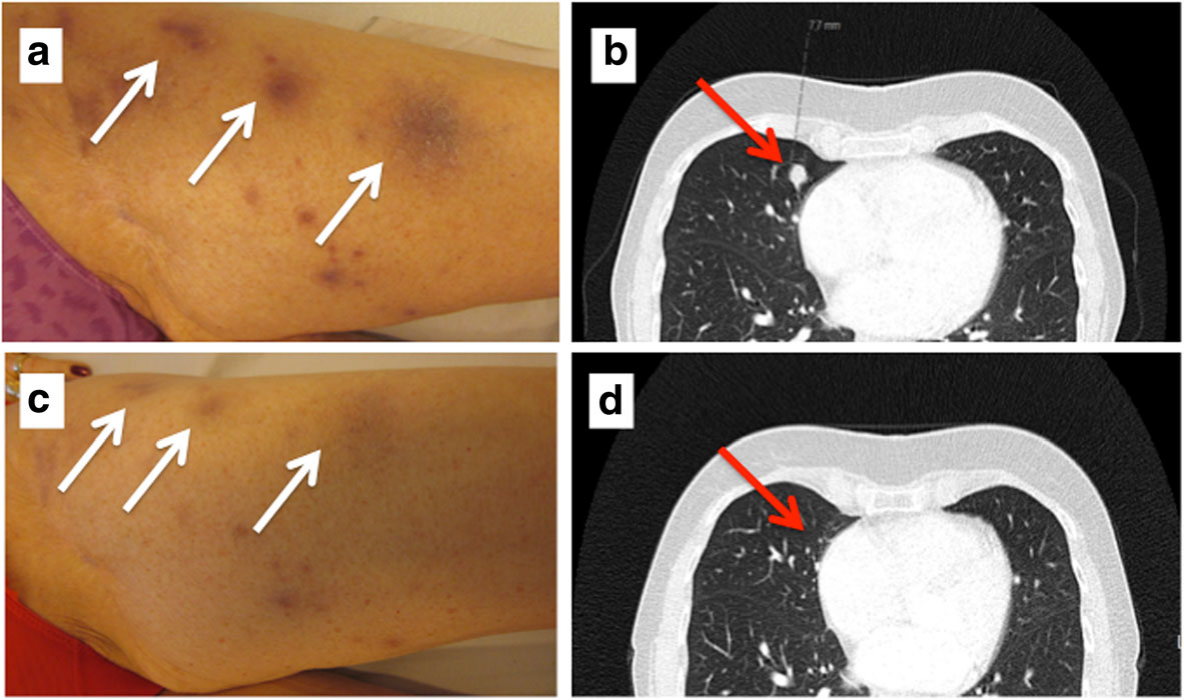
At recurrence the patient had multiple small metastases in both lungs (*red arrows*) and multiple subcutaneous nodules in her left groin and thigh (*white arrows*). There was no effect of chemotherapy and only 2 months’ effect of dabrafenib. Panels **a** and **b** show before treatment with ipilimumab and **c** and **d** after two cycles where a significant remission was seen. After four cycles she was completely tumor free, and still is with virtually no side effects

Due to the previous good results with chemotherapy, one course of paclitaxel and carboplatin was tried. However, the nodules grew more aggressively, and chemotherapy was discontinued in favor of a BRAF inhibitor, dabrafenib, that showed a brief response of 2 months, and subsequently vemurafenib was administered resulting in progressive disease. Combined treatment with a mitogen-activated protein kinase (MEK) inhibitor was not introduced in Norway at that time point. In the meantime, ipilimumab was approved for use in melanomas in Norway and in December 2014 she was offered this treatment. After four cycles of ipilimumab there was a complete response in her skin and her lungs, with no reported side effects (Fig. 4).

**Fig. 5:**
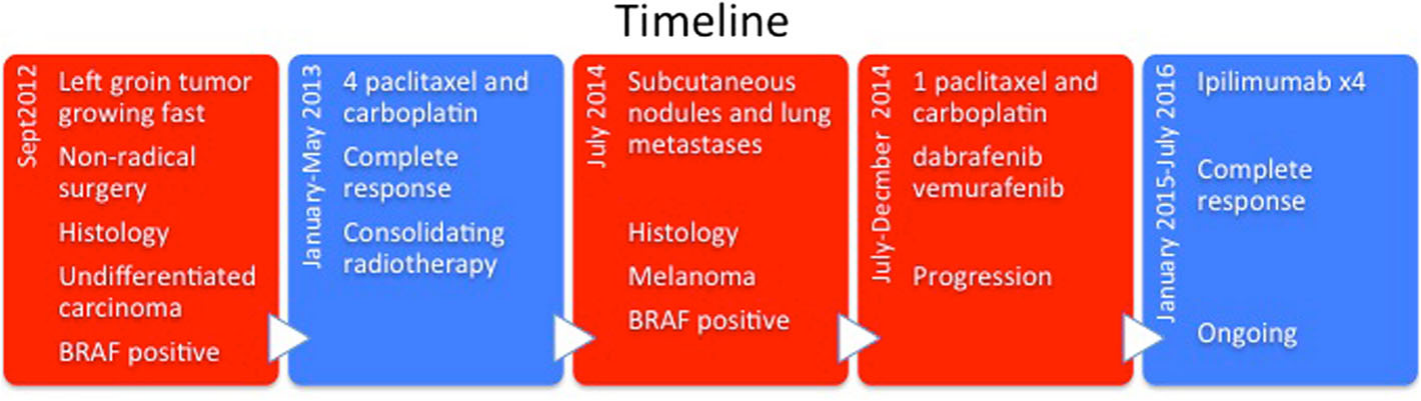
Timeline of the key events

Today, 4 years and 4 months after primary diagnosis of aggressive CUP and 2 years and 6 months after metastatic melanoma was diagnosed in the re-biopsy, she is radiologically and clinically cancer-free (Fig. 5).

## Discussion

Diagnosis is often difficult in CUP. On microscopic examination of the first resected left groin tumor that was a lymph node metastasis, malignant melanoma was excluded due to the immune profile with CK AE1/AE3 positivity and negative reaction for three melanoma markers. Due to this finding we re-stained the primary biopsy with S100, as well as HMB45 and melan A. All three markers were negative. Of interest, the few scattered S100 positive cells with dendritic features were also positive for CD68, and thus believed to represent tumor-associated macrophages.

Malignant melanomas, especially metastases, are known for aberrant immunohistochemical features in some cases. Expression of various non-melanoma markers, including intermediate filaments and loss of classical melanoma markers is not unusual [8, 9] and awareness of the possibility of unusual immunophenotypes is crucial for the right diagnosis. BRAF mutations are most commonly associated with malignant melanomas, colorectal adenocarcinomas, and papillary thyroid carcinoma and could therefore be helpful in identifying the origin of the tumor [10, 11]. Neither the clinical picture nor the histopathological examination supported the two last diagnoses. Moreover, the metastasis localized in the surgical field where the primary lymph nodes had infiltrated the skin, also indicated that this was the same tumor. In hindsight, the simultaneous finding of a BRAF mutation, combined with the localization and morphology of the tumor, should have aroused our suspicion of an aberrant malignant melanoma. Thus we conclude that in our case, the undifferentiated CUP probably was transformed to, or at least acquired the molecular characteristics of, a melanoma at recurrence, and was successfully treated with immune checkpoint therapy.

Metastatic melanoma was treated with chemotherapy in general until recently when the use of BRAF, MEK, CTL-4, and PD-1 inhibitors showed increased survival for patients, even beyond 5 years in a subset of patients [12, 13]. A predictive marker for BRAF inhibitors in melanoma is the BRAF V600E mutation, where positive tumors have a high response rate with increased progression-free survival for patients and when combined with a MEK inhibitor there is increased overall survival [14, 15]. Positive predictive markers for PD-1 inhibition in melanoma appear to be positive PD-L1 expression in a tumor [16] and low expression is predictive for combined PD-L1/CTL-4 inhibition. Moreover, checkpoint inhibitors seem to be very effective in several types of tumors with a high mutational burden as well as in tumors with microsatellite instability and/or mismatch repair deficiency [17]. In our patient, neither PD-L1 expression nor microsatellite status was evaluated. However, in the future, given the possible therapeutic implications, this may well become part of a future biomarker panel in both CUP and melanoma.

From the patient’s perspective, the decision to re-biopsy this recurrent CUP turned out to become a life-saving procedure that changed her future.

## Conclusions

Currently, with the advent of targeted treatment and immunotherapy, identifying molecular subtypes may be of benefit for several cancer types. Immunotherapy, by ipilimumab, a fourth-line treatment, induced a complete and durable response after recurrence and progression on chemoradiotherapy and two BRAF inhibitors. Moreover this patient had clinical benefit with paclitaxel-carboplatin on what was perceived as an undifferentiated carcinoma, which became resistant when transformed to a S100-positive melanoma.

In conclusion, molecular screening in the primary biopsy and re-biopsy of the recurrence may be of clinical value in CUP, where evaluations with a broad spectrum of immunohistochemical and molecular analyses are necessary.
